# Effector Tc17 cells resist shift from OXPHOS to aerobic glycolysis

**DOI:** 10.3389/fimmu.2025.1571221

**Published:** 2025-05-16

**Authors:** Reni John, Srinivasu Mudalagiriyappa, Nagabhushan Chandrashekar, Som G. Nanjappa

**Affiliations:** ^1^ Department of Pathobiology, College of Veterinary Medicine, University of Illinois Urbana-Champaign, Urbana, IL, United States; ^2^ Cancer Center at Illinois, University of Illinois at Urbana-Champaign, Urbana, IL, United States

**Keywords:** CD8+ T cell, Tc17 cell, glycolysis, OXPHOS, T cell activation, antifungal, vaccine responses

## Abstract

IL-17A-expressing lymphocytes, including Tc17 cells, are instrumental in immunity, immunopathology, and autoimmunity. We have previously shown that experimental attenuated live fungal vaccine-induced Tc17 cells are stable, long-lived without plasticity, and necessary to mediate sterilizing immunity during CD4^+^ T cell deficiency, which poses higher susceptibility to fungal infections. Cell metabolism is integral for T cell homeostasis but the metabolic adaptations of Tc17 cells are poorly defined. In this study, we hypothesized that effector Tc17 cells adopt high energy-yielding metabolic pathways to form stable, long-lived memory cells *in vivo*. Using a mouse model of attenuated fungal vaccination, we found that effector Tc17 cells were metabolically highly active with higher proliferation and protein synthesis than IFNγ^+^ CD8^+^ T (Tc1) cells. Glucose was necessary for effector Tc17 cell expansion but with less dependency during the late expansion despite the active metabolism. Contrary to established dogma, we found that the effector Tc17 cells preferentially channeled the glucose to OXPHOS than glycolysis, which was correlated with higher mitochondrial mass and membrane potential. Inhibition of OXPHOS shrunk the Tc17 responses while sparing Tc1 cell responses. Tc17 cells actively relied on OXPHOS throughout the expansion period, resisting adaptation to aerobic glycolysis. Our data showed that the effector Tc17 cells predominantly utilize glucose for metabolism through OXPHOS rather than aerobic glycolysis. Our study has implications in vaccine design to enhance the efficacy and immunotherapeutics to modulate the immunity and autoimmunity.

## Graphical Abstract

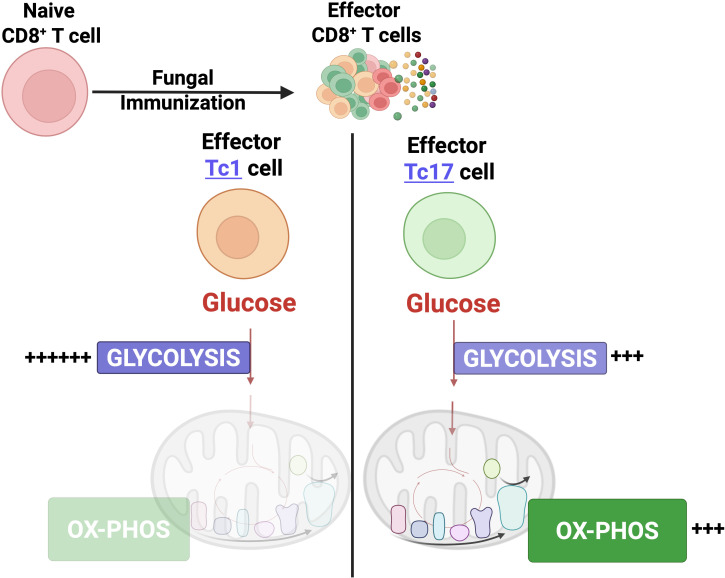

## Introduction

IL-17A-producing CD8^+^ T (Tc17) cells protect against viral, bacterial, and fungal infections and the homeostasis of commensal microbiome (1–4). Accumulating evidence implicates Tc17 cells in the pathology of autoimmune disorders like psoriasis, multiple sclerosis, and ulcerative colitis (5–7), including in pathologic pulmonary inflammations, such as allergic airway inflammation and smoke-induced emphysema (8, 9). Tumor-specific Tc17 cells are shown to be protective in eliciting immunity to melanoma (10) but are pathogenic in aggravating prognosis in GI tract-associated cancers (11). Thus, Tc17 cells play an instrumental role in immunity, immunopathology, and autoimmune disorders.

Tc17 differentiation largely follows the principles of Th17 differentiation. Following antigenic stimulation, under the lineage-promoting inflammatory micro milieu of IL-6, TGFβ, IL-1, and IL-23, naïve CD8^+^ T cells differentiate and expand into Tc17 cells in both *in vivo* and *in vitro (* 12). Previously, we have shown that fungal vaccine-induced Tc17 cells also required IL-6, IL-1β, and IL-23, and cell-intrinsic MyD88 signaling was required to enhance their responses (13–15). Tc17 shown to be long-lived compared with the IFN-γ producing CD8^+^ T (Tc1) subset, a feature employed for effective antitumor immunity (16). Although there is ample evidence of Tc17 cells’ plasticity towards IFN-γ or IL-5, and IL-13 (2, 17), our previous studies showed that *in vivo* differentiated Tc17 cells are long-lived and stable without plasticity towards IFN-γ-producing cells (15), suggesting contrasting features of *in vitro* polarized Tc17 cells with that of *in vivo*.

Metabolism is fundamentally integrated into T cell activation, differentiation, and function, and it is often considered to be Signal 4 (18, 19). The mammalian cells generate energy through two major catabolic pathways, glycolysis and mitochondrial oxidative phosphorylation (OXPHOS), and the dependency of these pathways differs during T cell homeostasis. Despite relatively low mitochondrial function, the quiescent naïve CD8^+^ T cells utilize oxidative phosphorylation for their sustenance and homeostatic proliferation (20–22). Activated CD8^+^ T cells metabolically reprogram to glycolysis with rapid ATP production, supporting their growth, proliferation, and effector functions (23). The naïve and memory cells depend on OXPHOS and fatty acid oxidation supported by glucose, where glucose was used to fuel mitochondrial FAO and OXPHOS (24). Although activated CD8^+^ T cells are known to utilize glycolysis, recent studies have shown that physiologically activated CD8^+^ T cells display different metabolisms than their *in vitro* counterparts (25), suggesting an apparent discrepancy in metabolic dependence of T cells under *in vitro* and *in vivo* conditions.

We have previously shown that experimental fungal vaccine induced Tc17 cells are long-lived, stable without undergoing plasticity, and mediate vaccine immunity against fungal infections during CD4^+^ T cell deficiency (13, 15). Despite the extensive but evolving understanding of metabolic adaptations of CD8^+^ T cells during immune responses, the metabolic adaptations by Tc17 cells are poorly understood. In this study, using a mouse model of fungal vaccine responses, we dissected the nature of metabolic reliance of effector Tc17 cells that may portend the longevity of Tc17 cells. We assessed the extent of glucose need during the expansion phase and assessed the relative contribution of OXPHOS for Tc17 cells contrasting with the Tc1 cells.

## Methods

### Mice

C57BL/6 mice were obtained from Jackson Laboratories (Bar Harbor, ME) or Charles River (Wilmington, MA). Breeding pairs of IL17a^tm1.1(icre)Stck^/J (Stock 016789) and B6.129X1-Gt(ROSA)26Sor^tm1(EYFP)Cos^/J (Stock 006148) were purchased from Jackson Laboratories and were intercrossed to maintain a heterozygous IL-17A^cre^ locus to analyze eYFP reporter and IL-17A protein expression. C.129S4(B6)-IFNg^tm3.1Lky/J^ (GREAT) mice were obtained from Jackson Laboratories (Bar Harbor, ME). Six-to-eight-week-old male and female animals were used for all the experiments. Animal breeding, housing, and execution of experiments were performed following the strict guidelines of the Institutional Animal Care and Use Committee at the University of Illinois Urbana-Champaign.

### Ethics statement

All animal procedures were performed according to the recommendations in the Guide for the Care and Use of Laboratory Animals of the National Institute of Health. Care was taken to minimize animal suffering. The animal work was done with the approval of IACUC at the University of Illinois Urbana-Champaign.

### Fungal culture, vaccination, and CD4^+^ T cell depletion

The isogenic strain of *Blastomyces dermatitidis* lacking BAD1 (Strain #55) was kindly provided by Bruce Klein (University of Wisconsin-Madison). The strain was grown on Middlebrook 7H10 agar slants supplemented with oleic acid-albumin complex (Sigma-Aldrich) maintained at 39°C in a humidified incubator. The mice were vaccinated subcutaneously (∼1 x 10^5^ CFU of strain # 55) at the dorsal and base of the tail regions. A weekly dose of 100µg of GK1.5 mAb (BioXCell, West Lebanon, NH) was administered by intravenous route to deplete CD4^+^ T cells with an efficiency of >95% depletion (26).

### Antibodies, surface markers and cytokine staining, and flow cytometry

The single-cell suspensions were prepared from the harvested skin-draining lymph nodes (dLN) and spleens by processing on 70μm strainers (BD Biosciences) on indicated days, and red blood cells were lysed using RBC lysis buffer (4% NH_4_Cl in PBS). To assess cytokine-producing CD8^+^ T cells, the single-cell suspensions from the tissues were re-stimulated with anti-mouse CD3e (Clone 145.2C11;0.1 mg/ml) and CD28 (Clone 37.51; 1 mg/ml) antibodies in the presence of Golgi-stop (BD Biosciences) at 37°C for 5 hrs. Cells were washed and incubated with anti-CD16/32 (Fc Block) antibodies for 15 min before staining for phenotypic markers in 2% BSA/PBS buffer along with the fixable live/dead stain (Invitrogen) for 30 mins on ice before staining for intracellular staining using a Fixation/Permeabilization Kit (BD Biosciences). All flow cytometry antibodies/reagents were purchased from BD Biosciences, Biolegend, Abcam, R&D Biosciences, or Invitrogen. The antibodies used in this study are: anti-mouse CD8a BV711 (Clone 53-6.7; Cat #563046), anti-mouse IL-17A AF647 (Clone Tc11-18H10; Cat #560184), anti-mouse IFN-γ AF700 (Clone XMG 1.2; Cat #557998), and anti-mouse TNF PE-Cy7 (Clone MP6-XT22,; Cat #557644) were purchased from BD Biosciences; anti-mouse CD44 BV786 (Clone IM7; Cat#103058), anti-mouse GM-CSF PerCP-Cy5.5 (Clone MPI-22E9; Cat#505409), anti-Puromycin PE (Clone 2A4; Cat #381504), and anti-Ki67 (Clone 16A8, Cat#652422) were purchased from Biologend; anti-GLUT-1 AF647 (Clone EPR3915; Cat#Ab195020) was purchased from Abcam; and anti-GLUT-2 PE (Cat#FAB1440P) was purchased from R&D Systems. Live/Dead Fixable Near IR viability kit (Cat#L34994) was purchased from Invitrogen. Stained cells were analyzed by flow cytometry using a full-spectrum Cytek Aurora analyzer, and the data were analyzed using FlowJo v10.10.0 software (BD Biosciences).

### Assessment of antigen-specific cytokine-producing CD8^+^ T cells *in vitro*

Bone marrow (BM) cells were harvested from femurs and tibia of naive mice and cultured in RPMI media supplemented with 10% fetal bovine serum (FBS), 1% Strepto-Penicillin, 20 ng/ml of GM-CSF, 10 ng/ml of IL-4, and 50 mM β- mercapto ethanol at 37°C for six days. On day 7, BM dendritic cells (BMDCs) were collected, washed, and incubated with heat-killed *B.d.* #55 yeast for a day. In the following day, enriched naïve CD8^+^ T cells were added to the culture (1DC: 0.5yeast: 2 T cells) and incubated for an additional four days. Golgi-stop (BD Biosciences) was added at the last 5 hours of stimulation, and the cells were collected to analyze cytokine-producing CD8^+^ T cells. Stained cells were analyzed by a spectral flow cytometer, Cytek Aurora.

### Quantifying of CD8^+^ T cell proliferation

The thymidine analog, BrdU, was pulsed (0.8 mg/ml daily change) in drinking water. The single-cell suspensions from the tissues were surface and intracellular staining for cytokines before staining for anti-BrdU antibody staining (BrdU kit) according to the manufacturer’s instructions (BD Pharmingen). Alternative to BrdU staining, Ki-67 staining was performed in some experiments to assess CD8^+^ T cell proliferation. Briefly, the restimulated single-cell suspensions were surface and intracellular cytokine stained before incubating with 1X Lysing Solution (BD Biosciences) at room temperature (RT) for 10 min. Cells were washed and incubated with Permeabilizing Solution (BD Biosciences) at RT for 15 min before staining with anti-Ki-67 mAb. BrdU incorporation in DNA and Ki67 staining of cells were analyzed by flow cytometry.

### Inhibition of glycolysis

The glucose analog and glycolysis inhibitor, 2-deoxy glucose (2-DG), was purchased from Sigma (St.Louis, MO) and was re-suspended in 1X PBS. 2-DG was administered (250 mg/kg) BID intraperitoneally. On the following day of the last administration, dLNs and spleens were harvested and cytokine-producing CD8^+^ T cells were analyzed by spectral flow cytometry.

### Assessment of mitochondrial mass and mitochondrial membrane potential

On indicated days post-vaccination, the tissues were harvested, single-cell suspensions were restimulated for 4.5 hours and incubated with 100 nM MitoTracker Deep Red (Invitrogen) in RPMI media supplemented with 10% FBS and 1% P/S at 37°C for 30 min according to the manufacturer’s instructions. The mitochondrial membrane potential of cells was assessed using tetramethyl rhodamine, methyl ester (TMRE; BD Biosciences). Single-cell suspensions were incubated with TMRE (50nM) in complete RPMI media at 37°C for 30 min (48). Stained cells were analyzed by flow cytometry.

### Inhibition of oxidative phosphorylation

Oligomycin, an oxidative phosphorylation inhibitor, was purchased from Selleck Chemicals (Houston, TX) and re-suspended in DMSO according to the manufacturer’s instructions. On day 2 after the addition of CD8^+^ T cells, oligomycin (2uM) was added to the T cell culture. On day 5 post-culture, cells were collected to analyze cytokine-producing CD8^+^ T cells by flow cytometry.

### Scenith assay

Single-cell suspension cells in RPMI supplemented with 10% FBS and Penicillin/Streptomycin were plated at 1x10^6^ cells/ml in a 48-well plate. Cells were incubated with 2-deoxy glucose (50 mM), oligomycin (1 µM) or both at 37°C for 30 min. Later cells were incubated with puromycin (10 ug/ml; Sigma) for 30 min. Cells were washed, stained for cell surface markers and intracellularly with anti-puromycin mAb (Biolegend), and analyzed by flow cytometry.

### Statistical analysis

The statistical significance of differences with more than two groups was measured by parametric one-way ANOVA with Tukey’s multiple comparison test. All other statistical analysis was performed using a two-tailed unpaired student’s t-test. GraphPad Prism v10.1.1 (GraphPad Software, LLC) was used for all statistical analysis. The values shown in the figures are mean ± SD. A two-tailed p-value of ≤ 0.05 was considered statistically significant.

## Results

### Effector Tc17 cells are highly metabolically active cells

**Figure 1:**
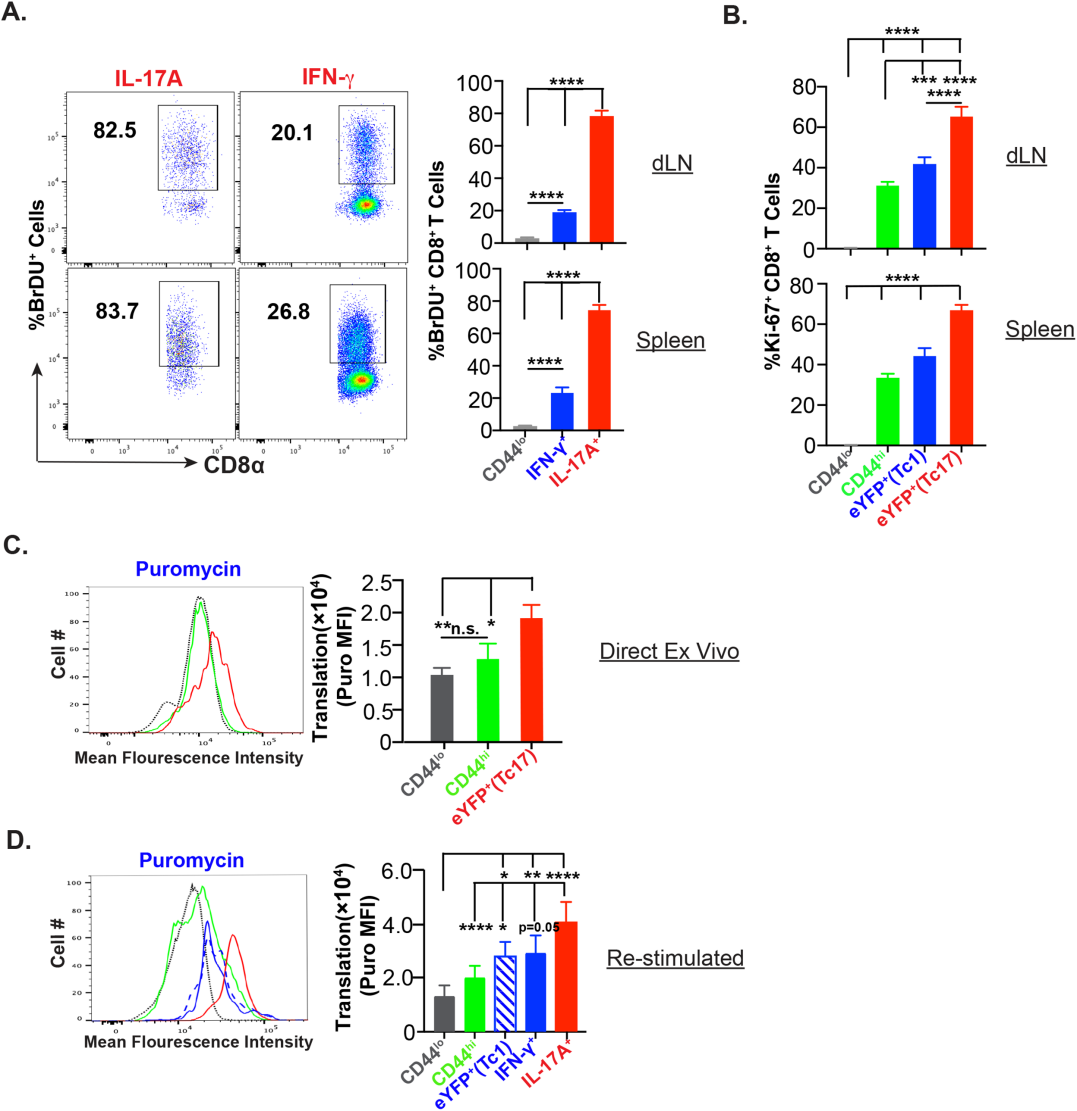
Effector Tc17 cells are metabolically active. **(A)** *Proliferation of anti-fungal effector CD8^+^ T cells by BrdU:* Naïve WT mice were vaccinated subcutaneously with ~10^5^ CFU of attenuated #55 strain of *B.dermatitidis (B.d. #55)* and were pulsed with BrdU (0.8 mg/ml) in drinking water from day 5 to 15 post-vaccination (PV). On day 16PV, draining lymph nodes (dLNs) and spleens were harvested to prepared single-cell suspensions and were restimulated with αCD3/CD28 mAbs. Cells were surface- and intracellular cytokine stained before BrdU staining. Numbers represent the percent BrdU^+^ among IL-17A^+^/IFN-γ ^+^ CD8^+^ T cells. **(B)** *Proliferation of effector CD8^+^ T cells by Ki67 staining*: Single-cell suspensions from dLNs and spleens of vaccinated IL17a^Cre^R26R^eYFP^ and GREAT mice were stained with anti-Ki-67 monoclonal antibody (mAb) intracellularly following surface markers and cytokine staining, and the frequencies of Ki-67^+^ cells were analyzed by flow cytometry. The numbers represent the percent Ki-67^+^ cells among naïve (CD44^lo^), activated (CD44^hi^), and IL-17A or IFN-γ eYFP^+^ CD8^+^ T cells **(C, D)** *Translational ability of CD8^+^ T cells*: Single cell-suspensions from dLNs of vaccinated IL17a^Cre^R26R^eYFP^ and GREAT mice were stained direct ex vivo or following restimulation with surface markers and intracellular staining following incubation with puromycin. Data show the mean fluorescence intensity of incorporated puromycin in naïve (CD44^lo^), activated (CD44^hi^) and IL-17A/IFN-γ^+^ eYFP^+^ CD8^+^ T cells. Values are mean ± SD. N=4–5 mice/group. Data are representative of ≥2 individual experiments. *p≤ 0.05, **p≤ 0.01, ***p≤ 0.001 and ****p≤ 0.0001. ns-not significant. p≥0.05.

Since effector cells need to reprogram metabolism due to rapid high energy demand for differentiation, proliferation, and survival, we first investigated the metabolic status of vaccine-induced effector Tc17 cells contrasting with naïve and IFN-γ (Tc1) producing cells. Tc17 cells were promptly expressed lineage-defining transcription factor, RORγt, and expressed IL-17A/IL-17F, where ~50% of the cells expressed both (**Supplementary Figure 1A**) following the gating strategy shown in the upper row panels (**Supplementary Figure 1**) In line with our previous publications, BrdU pulse experiments showed that Tc17 cells were robust in proliferation compared with Tc1 cells in draining lymph nodes, the site of vaccine-induced responses (**Figure 1A**). Higher degree of proliferation was sustained in the spleen even after emigrating from the site of draining antigens suggesting the imprinting of augmented proliferative potential of effector Tc17 cells. We confirmed this phenotype using IL-17A and IFN-γ reporter mice by direct ex vivo staining for Ki-67, a proliferation potency indicator (**Figure 1B**, **Supplementary Figure 1B**). Further, the higher proliferative potential is retained following the contraction phase in the early memory phase in Tc17 cell over Tc1 cells (**Supplementary Figure 1C**).

Protein translation is integral for the expression of functional proteins, cellular growth and division, and can be used to measure cellular metabolism. We used Scenith Assay, where puromycin integrated into newly synthesized proteins can be assessed by flow cytometry. Using IL-17 reporter mice, we profiled the metabolic status of effector Tc17 cells. As expected, naïve CD8^+^ T cells had relatively lower metabolic activity than the activated (CD44^hi^) cells (**Figure 1C**). However, eYFP^+^ CD8^+^ T cells (Tc17) showed significantly higher metabolic status than the naïve and CD44^hi^ CD8^+^ T cells. Since the puromycin staining requires fixing and permeabilization of cells, leading to loss of eYFP signal in GREAT (IFN-γ-reporter) mouse cells for direct ex vivo staining, we adopted restimulation before running the assay. We found that Tc17 cells had significantly higher metabolic status than the eYFP (Tc1) or IFN-γ (Tc1) cells (**Figure 1D**).

Collectively, Tc17 cells are metabolically highly active with robust proliferation and high rate of protein synthesis.

### 17 Effector Tc17 cells rely on glucose for their expansion

Unlike naïve T cells, the effector T cells preferentially utilize glucose for their rapid energy demand during the expansion phase (22). Glucose is an instrumental, readily available energy source preferentially used by T cells. Further, glycolytic pathway intermediates are used to synthesize nucleotides, proteins, and lipids for cellular growth and proliferation. First, we measured the expression of classic glucose transporters 1 and 2 (GLUT1/2) on eYFP^+^ CD8^+^ T cells in vaccinated IL-17A-reporter mice. The mean fluorescence intensity of GLUT1 and GLUT2 transporters (direct ex vivo) were significantly higher in Tc17 cells than in naïve T cells during mid- and late phases during expansion following vaccination (26) (**Supplementary Figure 2A**).

**Figure 2:**
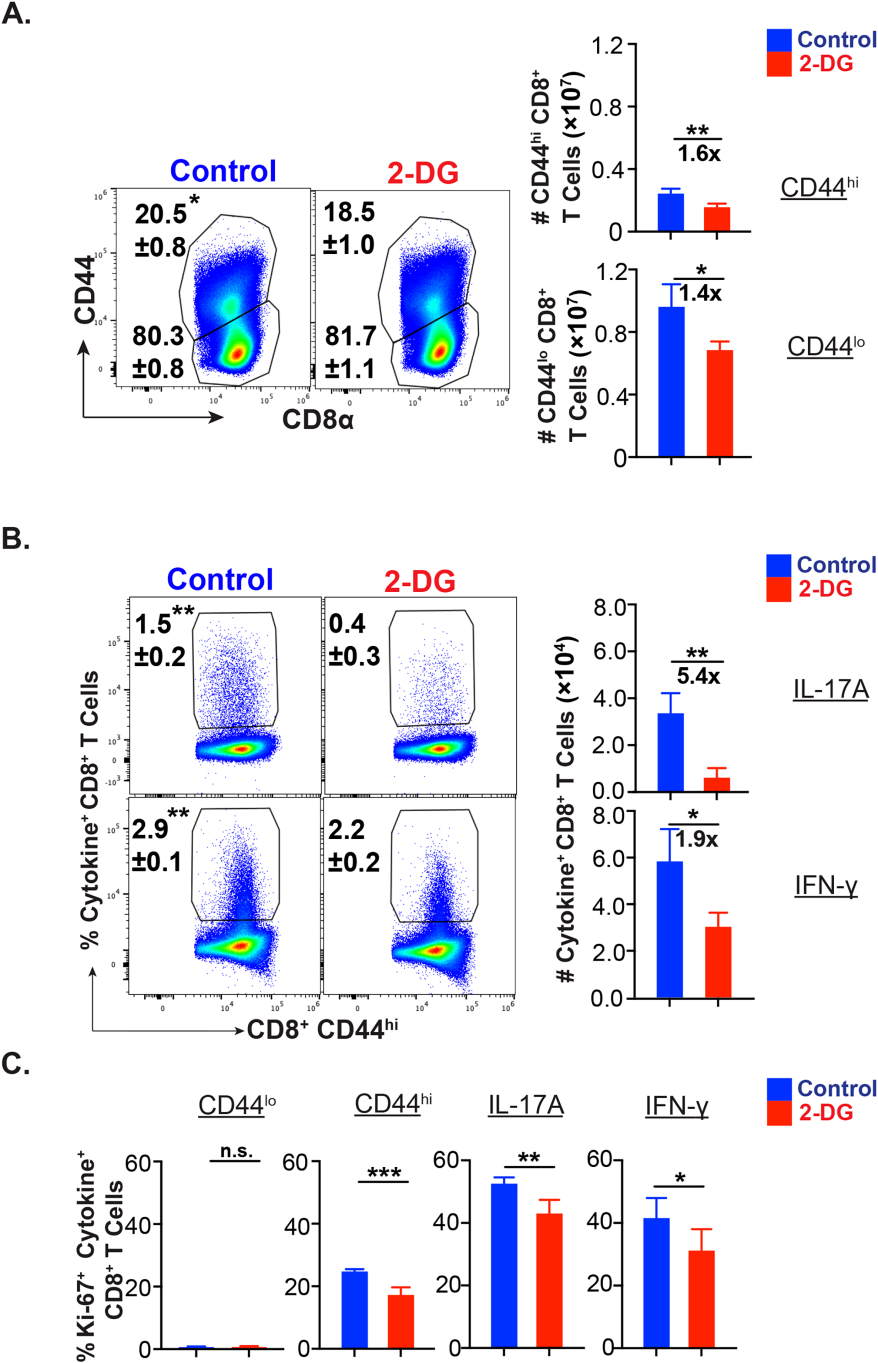
Glucose dependency of effector Tc17 cells. **(A-C)** *Glucose utilization of antifungal CD8^+^ T cells for activation, proliferation, and cytokine expression*: Naïve 6- to 8- week-old IL17a^Cre^R26R^eYFP^ mice were vaccinated with *B.d*. #55 (~ 10^5^ CFU) and treated with PBS or glycolysis inhibitor 2-deoxy glucose (2-DG) intraperitoneally from day 5 to 15 PV. On day 16PV, single-cell suspensions from dLNs were restimulated with αCD3/CD28, surface and intracellular stained, and analyzed by flow cytometry. Data represent percent and total numbers of CD44^lo^ and CD44^hi^ cells gated on CD8^+^ T cells. **(A)** and IL-17A^+^/IFN-γ ^+^ cells gated on CD44^hi^ CD8^+^ T cells **(B)** and percent of Ki-67^+^ cells among CD44^lo^, CD44^hi^, IL-17A^+^ and IFN-γ^+^ CD8^+^ T cells **(C)**. Values are in mean ± SD. Data are representative of two independent experiments. n = 4–5 mice/group. *p≤ 0.05, and **p≤ 0.01.

Next, we asked about relative dependency of effector CD8^+^ T cells on glucose during the mid-expansion phase *in vivo*. The administration of glucose analog, 2-deoxy-glucose (2-DG), significantly reduced the frequency of activated (CD44^hi^) but not the naïve (CD44^lo^) CD8^+^ T cells (**Figure 2A**), confirming the increased need for glucose following the activation of naïve CD8^+^ T cells. The administration of 2-DG significantly reduced cytokine-producing cells (**Figure 2B**; **Supplementary Figure 2B**). However, the dependency on glucose was significantly higher for effector Tc17 cells than the Tc1 cells, and 2-DG administration reduced the Tc17 cells by ~54% in contrast to the ~19% reduction of Tc1 cells (**Figure 2B**). Similarly, the proliferation of Tc17, Tc1, and activated CD8^+^ T cells, measured by Ki67 staining, was significantly reduced following 2-DG administration (**Figure 2B**).

Thus, effector Tc17 cells need glucose for their metabolic needs during the expansion phase of CD8^+^ T cell responses *in vivo*.

### Effector Tc17 cells need glucose in the early but not late phase of expansion

**Figure 3:**
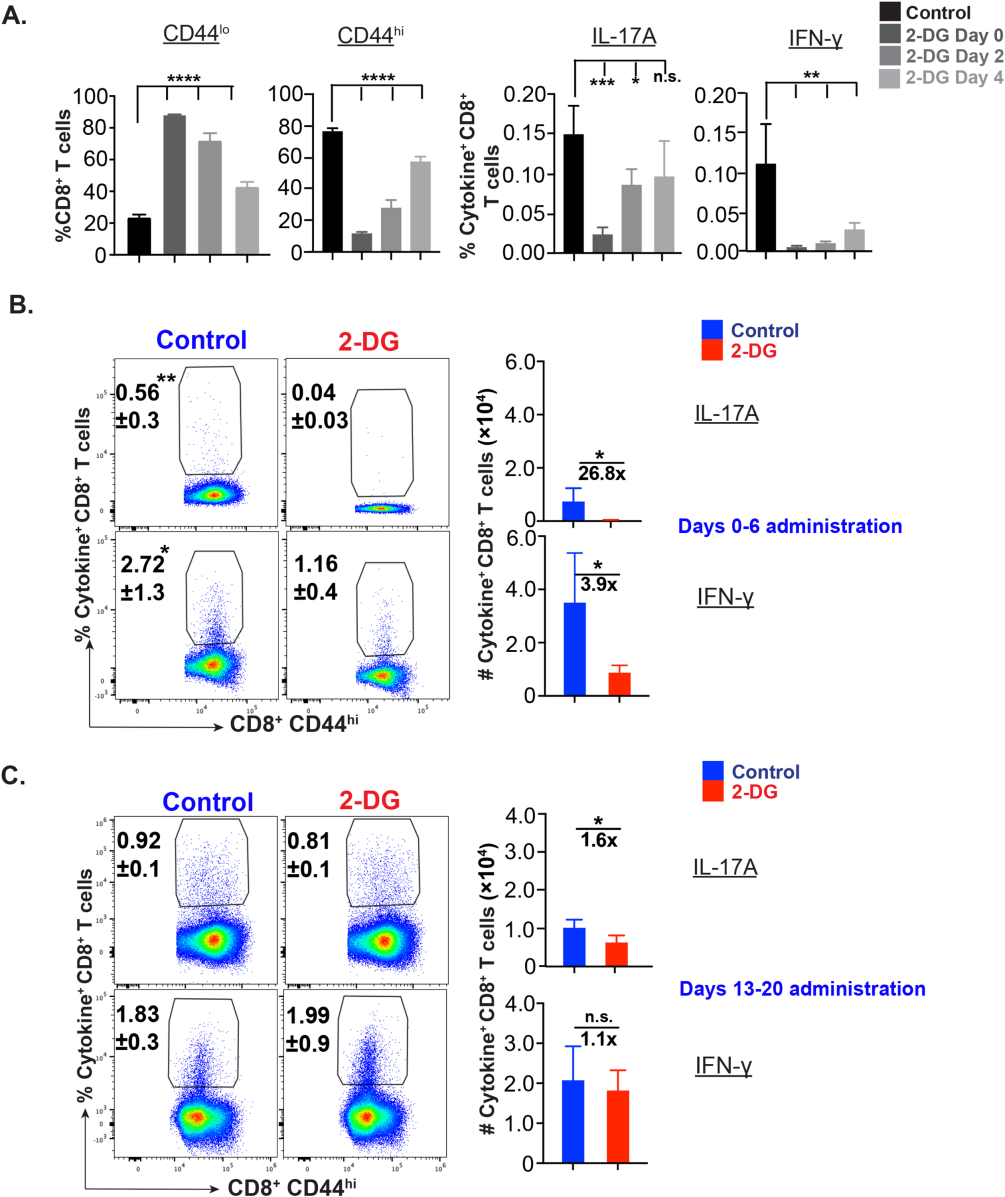
Glucose dependency of effector CD8^+^ T cells during early and late expansion. **(A)** *Phase* sp*ecific glucose dependency of antifungal CD8^+^ T cells in vitro*: ^©^ CD8^+^ T cells (WT mice) were enriched and cultured with bone marrow-derived dendritic cells pulsed with heat-killed yeast. On days 0-4, 2-4, and 4 post-culture, cells were treated with 250 µM 2-deoxy glucose. The cells were harvested on day 5 post-culture and stained for cytokine^+^ CD8^+^ T cells. Data show the percent naïve, activated, IL-17A^+^, and IFN-γ^+^ cells gated on (CD44^hi^) CD8^+^ T cells. **(B, C)** *Glucose dependency of effector CD8^+^ T cells in vivo*: Vaccinated were administered with vehicle or 2-deoxy glucose (2-DG) intraperitoneally on indicated days. On days 7 or 21 post vaccination (PV), single-cell suspensions from dLNs were restimulated with αCD3/CD28 mAbs, surface and intracellular cytokine-stained, and analyzed by flow cytometry. Data show percent and total numbers of IL-17A^+^ and IFN-γ^+^ cells gated on CD44^hi^ CD8^+^ T cells on days 7 PV (days 0–6 PV administration; **(B)**) and 21PV (days 13–20 PV administration; **(C)**). Data are representative of two independent experiments, n= 5–6 replicates/group **(A)** and n= 4–5 mice/group **(B, C)**. Values are mean ± SD. *p≤ 0.05, **p≤ 0.01, ***p≤ 0.001, and ****p≤ 0.0001. ns-not significant , p≥0.05.

To further delineate the glucose requirement during the expansion phase, we leveraged on a recently lab-developed *in vitro* assay to determine the fungal-specific CD8^+^ T cells. Incubation with 2-DG for various durations differentially affected the frequency of cytokine-producing CD8^+^ T cells with reduced effect in the later phase, especially on Tc17 cells (**Figure 3A**). Previously, we have shown that the immunization of mice with live attenuated yeasts induces efficient CD8^+^ T responses peaking around day 21 post-vaccination (26). To assess glucose dependency of effector CD8^+^ T cells during the different phases of expansion *in vivo*, we pulsed 2-DG in the early (0–6 day post-vac) and late (13–20 day post-vac) expansion phase to contrast with the mid-expansion (**Figure 2**). The administration of 2-DG during the early phase of expansion significantly decreased the cytokine-producing cells as compared with administration during the late phase (**Figures 3B, C**) despite modest effects on activated CD8^+^ (CD44^hi^) T cells (**Supplementary Figures 3A, B**). The percent reduction of Tc17 cells during the early phase of expansion period was higher than IFN-γ^+^ CD8^+^ T cells. Notably, like *in vitro* data (**Figure 3A**), the Tc17 cells were less prone to glucose starvation in the late phase of expansion (**Figure 3C**).

Thus, effector Tc17 cells are highly dependent on glucose in the early phase of expansion, and glucose dependency wanes during the last phase of expansion.

### Effector Tc17 cells have high mitochondrial mass and potential

**Figure 4:**
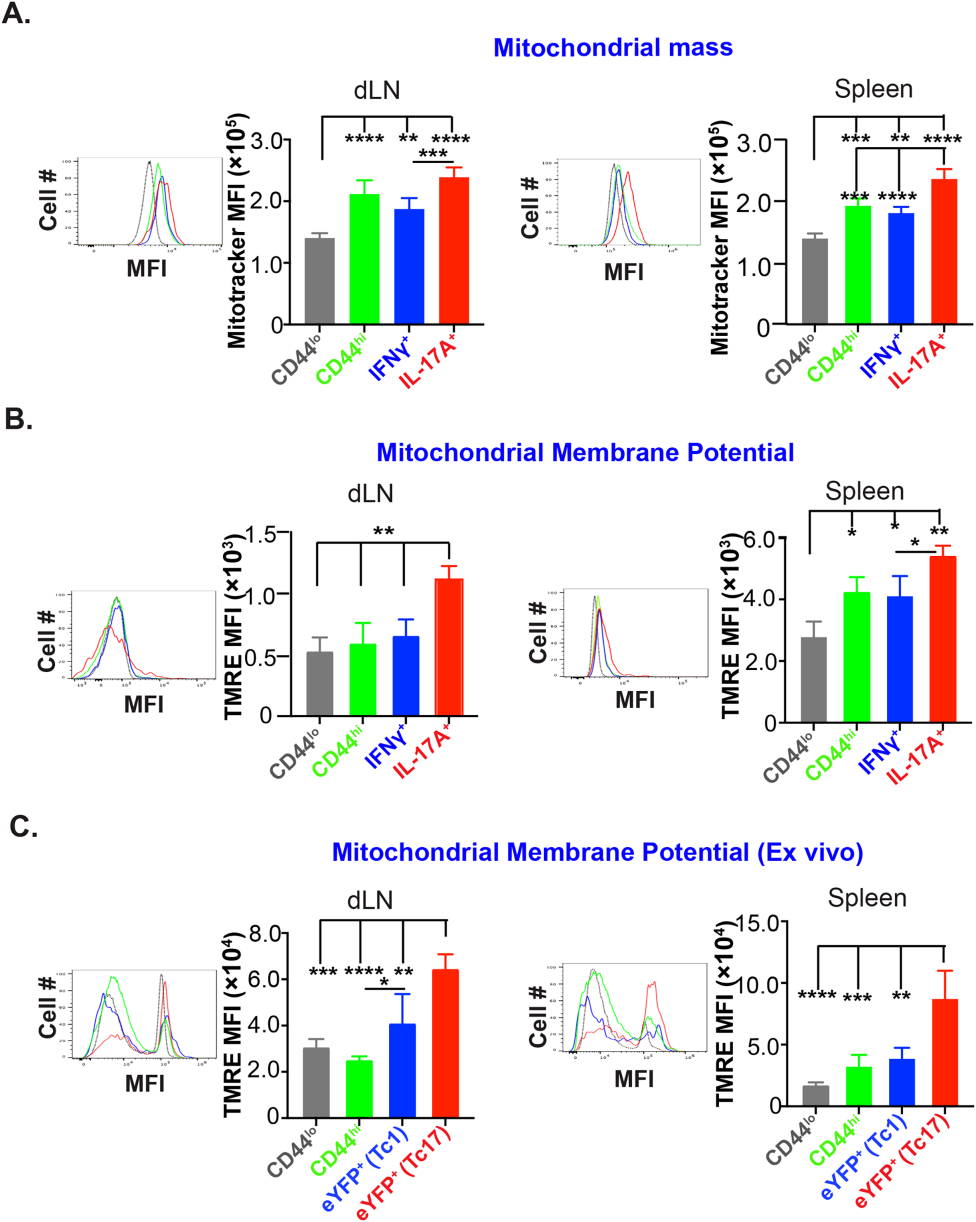
High mitochondrial mass and membrane potential of effector Tc17 cells. **(A)** *Mitochondrial mass*: Naïve WT mice were vaccinated with *B.d.* #55. On day 16 post-vaccination (PV), mitochondrial mass of CD8^+^ T cells of draining lymph nodes (dLNs) and spleens was assessed by MitoTracker Deep Red staining and analyzed by flow cytometry. Data show the mean fluorescence intensities of MitoTracker Deep Red in CD44^lo^, CD44^hi^, IL-17A^+^, and IFN-γ^+^ CD8^+^ T cells. **(B)** *Mitochondrial membrane potential*: Single-cell suspensions from vaccinated mice (day 16PV) were restimulated (anti-CD3/CD28 mAbs), incubated with Tetramethyl Rhodamine, Ethyl Ester (TMRE), before staining for cell surface markers and intracellular cytokines for flow cytometry. Data show the mean fluorescence intensities of TMRE in CD44^lo^, CD44^hi^, IL-17A^+^, and IFN-γ^+^ CD8^+^ T cells. **(C)** *Mitochondrial membrane potential of effector CD8^+^ T cells from reporter mice*: Single-cell suspensions from dLNs and spleens of vaccinated IL17a^Cre^R26R^eYFP^ and GREAT mice (on day 21PV) were incubated with TMRE before staining for cell surface markers and analyzed by flow cytometry. Data show the mean fluorescence intensities of TMRE in naïve, activated, and eYFP^+^ CD8^+^ T cells. Values are mean ± SD. Data are representative of two independent experiments. n= 3–5 mice/group. *p≤ 0.05, **p≤ 0.01, ***p≤ 0.001, and ****p≤ 0.0001.

Although effector Tc17 cells reduced their glucose dependency during late expansion, Tc17 cells are still highly metabolically active during late/peak of the fungal vaccine response, i.e., ~3-wk post-vaccination (**Figure 1**) (26). It is documented that the activated T cells shift the metabolism towards aerobic glycolysis, which rapidly produces ATP and provides glycolytic intermediates for biosynthesis. However, the lactate production pathway may be reduced if glycolysis intermediates enter the biosynthetic pathway. Recent evidence suggests that the activated T cells can considerably upregulate OXPHOS, a process that surpasses aerobic glycolysis in terms of energy production efficiency (27). Apart from ATP production, mitochondria are a hub for signaling, apoptosis, cell cycle progression, calcium homeostasis and lipid synthesis. Mitochondria adapt to these functions by undergoing biogenesis, fission, fusion, and mitophagy. Thus, to elucidate the role of mitochondria for energy generation in glucose-deprived conditions in effector Tc17 cells, we evaluated the changes in the numbers and function of mitochondria during the expansion phase. First, we measured the mitochondrial mass of the effector CD8^+^ T cells using MitoTracker dye. Mitochondrial mass in activated (CD44^hi^) CD8^+^ T cells was significantly higher than the naïve counterparts but similar to IFN-γ expressing CD8^+^ T cells in dLN and spleens (**Figure 4A**). However, IL-17A expressing CD8^+^ cells had significantly higher mitochondrial mass than Tc1 cells suggesting a significant increase in the mitochondrial biogenesis. Next, we asked if higher mitochondrial mass corresponds to higher mitochondrial activity in effector Tc17 cells. Mitochondrial membrane potential is inevitable in a cell’s oxidative phosphorylation, leading to efficient ATP generation, and could be used to assess the functional status (28). We used tetramethyl rhodamine ester (TMRE) to measure the mitochondrial membrane potential of effector CD8^+^ T cells (29). Again, Tc17 cells showed higher mitochondrial membrane potential than naïve, activated CD8^+^ T cells and Tc1 cells in dLN and spleen (**Figure 4B**). To exclude the possibility of enhanced mitochondrial potential of Tc17 cells due to ex vivo restimulation, we used IL-17A and IFN-γ reporter mice for vaccination to assess mitochondrial potential by direct ex vivo. Again, we found that the effector Tc17 cells had higher mitochondrial membrane potential than Tc1 cells in dLN and spleens (**Figure 4C**), suggesting their superior mitochondrial activity.

Thus, effector Tc17 cells have high mitochondrial mass and portray higher mitochondrial activity.

### Effector Tc17 cells adopt oxidative phosphorylation

**Figure 5:**
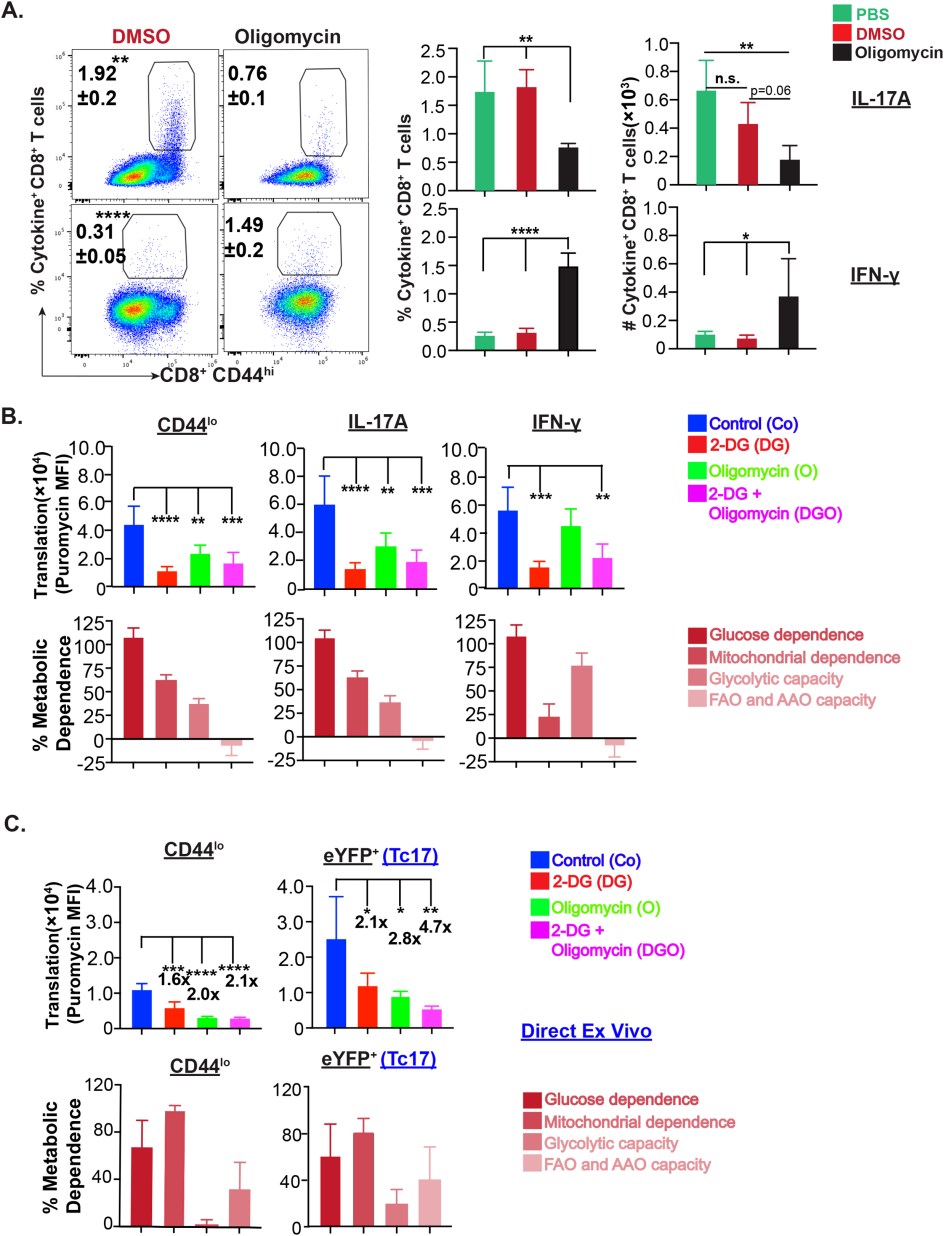
Mitochondrial metabolism by effector Tc17 cells. **(A)** *OX-PHOS utilization by antifungal effector CD8^+^ T cells in vitro. *Naive CD8^+^ T cells were enriched and cultured with bone marrow-derived dendritic cells (BMDCs) pulsed with heat-killed yeast. On days 2–4 culture, cells were treated with vehicle or Oligomycin. The cells were harvested on day 5 post-culture, stained for surface markers and intracellular cytokines, and analyzed by flow cytometry. Data show the percent and total numbers of IL-17A^+^ and IFN-γ^+^ cells gated on CD44^hi^ CD8^+^ T cells. **(B)** *Metabolic dependence of effector CD8^+^ T cells:* Single-cell suspensions from dLNs of vaccinated GREAT mice (day 21 PV) were restimulated with αCD3/CD28 mAbs before treating with vehicle, 2-deoxyglucose (2-DG), oligomycin or both for 30 min followed by incubation with puromycin for 30 min. Cells were stained for cell surface markers and intracellular cytokine and puromycin and analyzed by flow cytometry. Data show the mean fluorescence intensities of puromycin incorporation in naïve, IL-17A^+^, and IFN-γ^+^ CD8^+^ T cells. **(C)** *Metabolic dependence of IL-17A reporter effector CD8^+^ T cells:* Single-cell suspensions from dLNs from vaccinated IL17a^Cre^R26R^eYFP^ mice (day 21PV) were treated with vehicle, 2-deoxyglucose (2-DG), oligomycin or 2-DG and oligomycin for 30 min followed by incubation with puromycin for 30 min. Cells were stained for cell surface markers and intracellular puromycin incorporation and analyzed by flow cytometry. Data show the mean fluorescence intensities of puromycin in naïve and eYFP^+^ CD8^+^ T cells. Values are mean ± SD. Data are representative of two independent experiments, n= 5–6 replicates/group **(A)** and n= 4–5 mice/group **(B, C)**. *p≤ 0.05, **p≤ 0.01, ***p≤ 0.001 and ****p≤ 0.0001. ns-not significant , p≥0.05.

We next asked whether the active mitochondria correlate with OXPHOS utilization and are necessary for Tc17 cell expansion. We utilized inhibitor oligomycin, which inhibits F0/F1 ATP synthase present across the inner mitochondrial membrane and generates ATP using the proton gradient generated by the electron transport chain. As expected, OXPHOS inhibition profoundly reduced the survival of naïve CD8^+^ T cells, which they primarily rely on (30) (**Supplementary Figure 4A**). Although the frequency of activated CD8^+^ T cells were unaffected in oligomycin treated group compared to control groups, their numbers were modestly reduced (**Supplementary Figure 4A**). Similarly, OXPHOS inhibition significantly reduced the frequency and numbers of Tc17 but not the Tc1 cells (**Figure 5A**). Since glucose was required for Tc17 responses (**Figure 2**) and OXPHOS inhibition reduced the Tc17 cells, we asked the relative dependency of Tc17 cells on glycolysis compared with mitochondrial dependence. We leveraged on Single Cell Energetic metabolism by profiling Translation inhibition (SCENITH) assay, in which metabolic capacities and dependencies can be measured with smaller number of effector cells (31). As expected, naïve and non-differentiated activated CD8^+^ T cells had lesser active metabolism indicated by low puromycin incorporation compared with effector CD8^+^ T cells and were mostly dependent on mitochondria (**Figure 5B**, **Supplementary Figure 4B**). Effector (both IL-17A and IFN-γ) CD8^+^ T cells significantly reduced the puromycin incorporation when 2-DG was added, suggesting the necessity of glucose for their metabolic energy. Notably, IL-17A^+^ but not IFN-γ^+^ CD8^+^ T cells were significantly less positive for puromycin incorporation in the oligomycin treated group and were dependent on mitochondrial function (OXPHOS) more than the glycolysis. Since ex vivo restimulation may have inadvertent effects and may switch to glycolysis, we pursued reporter mice for direct ex vivo assay to analyze puromycin incorporation. We found that the phenotype was recapitulated by the predominant dependence of Tc17 cells on mitochondria more than glycolysis, and glucose was a major driving force (**Figure 5C**).

Thus, effector Tc17 cells primarily rely on glucose-driven oxidative phosphorylation.

### Effector Tc17 cells utilize glucose for OXPHOS during early expansion

**Figure 6:**
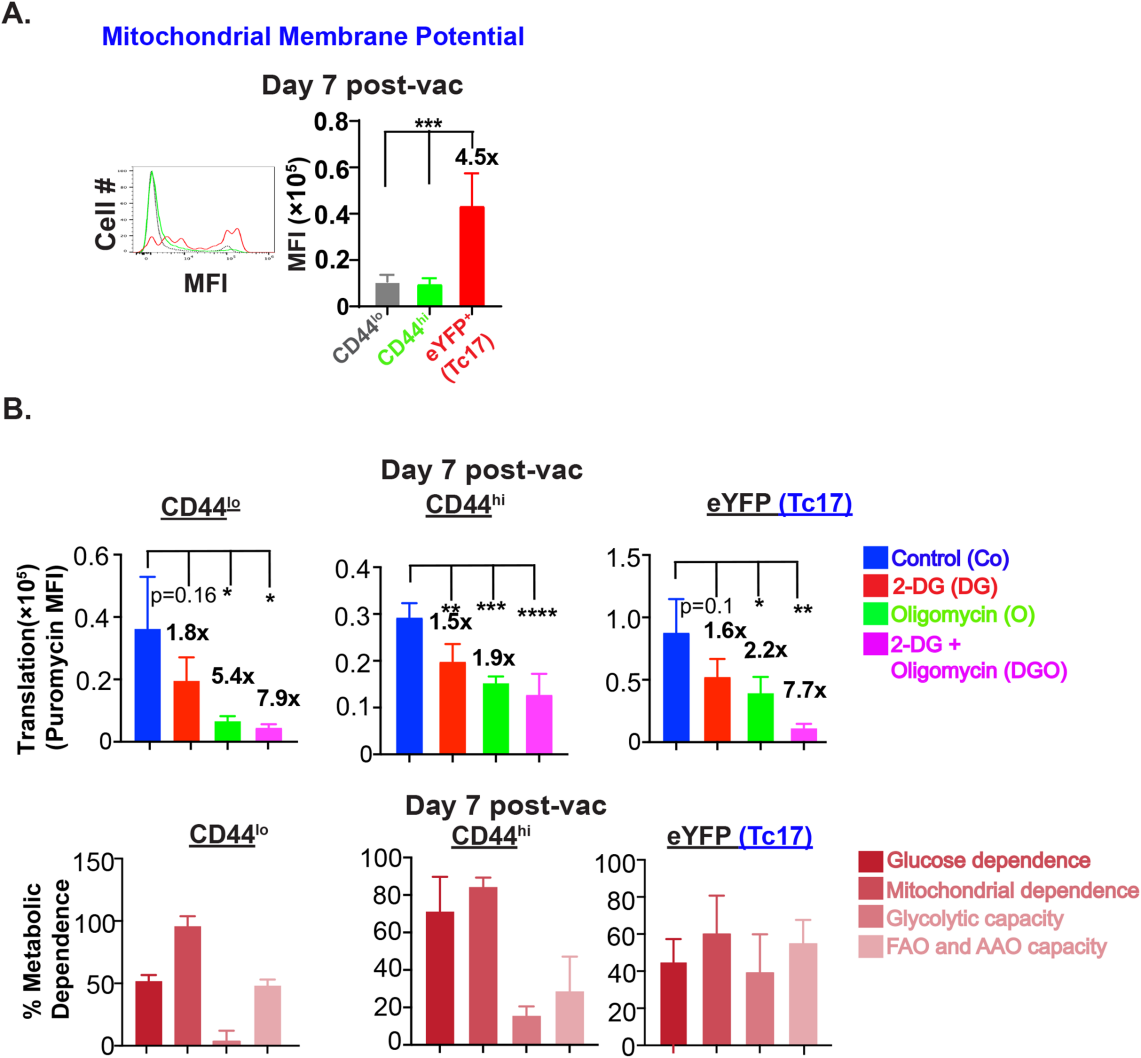
Glucose for OX-PHOS by Tc17 cells during early expansion. **(A)** Mitochondrial membrane potential of IL-17A reporter effector CD8^+^ T cells: Single-cell suspensions from dLNs of vaccinated IL17a^Cre^R26R^eYFP^ mice (on day 7 PV) were incubated with 50 nM tetramethyl rhodamine, ethyl ester (TMRE) for 30 min followed by staining for cell surface markers. Stained cells were analyzed by flow cytometry. Data show the mean fluorescence intensities of TMRE in naïve, activated, and eYFP^+^ CD8^+^ T cells. **(B)** *Metabolic dependence of effector CD8^+^ T cells*: Single-cell suspensions from dLNs of vaccinated IL17a^Cre^R26R^eYFP^ mice (on day 7 PV) were treated with vehicle, 2-DG, oligomycin or both 2-DG and oligomycin for 30 min followed by incubation with puromycin for 30 min. Cells were stained for cell surface markers and intracellular puromycin and analyzed by flow cytometry. Data show the mean fluorescence intensities of puromycin incorporation and percent metabolic dependence of naïve, activated, and eYFP^+^ CD8^+^ T cells. Values are mean ± SD Data are representative of two independent experiments. n= 4–5 mice/group. *p≤ 0.05, **p≤ 0.01, ***p≤ 0.001 and ****p≤ 0.0001.

The aforementioned data suggested that Tc17 cells are utilizing glucose-fueled OXPHOS *at the peak* of the effector response that befitted the phenotype of considerably less dependency on glucose (**Figure 3C**). Since aerobic glycolysis is necessary for T cell activation (32), next, we asked if Tc17 cells rely on OXPHOS or glycolysis early in the expansion. First, we assessed the mitochondrial potential at day 7 post-vaccination. Interestingly, Tc17 cells showed significantly higher mitochondrial function early in the expansion phase (**Figure 6A**). Next, we evaluated the metabolic capacity and dependency of Tc17 cells at day 7 using the SCENITH assay. Early effector Tc17 cells were dependent on mitochondria and showed glucose-driven OXPHOS and glycolysis with support from FAO and AAO (**Figure 6B**). We further examined the cell-intrinsic role of OXPHOS for Tc17 cell responses using *in vitro* T cell-polarization. Our data suggested that addition of oligomycin reduced the Tc17 cell responses and was dependent on the glucose (**Supplementary Figure 4C**).

Thus, effector Tc17 cells resist metabolic shift from mitochondrial dependency to aerobic glycolysis during expansion phase.

## Discussion

Type 17 cells provide immunity to many fungal and bacterial infections and are implicated in autoimmune disorders. Tc17 cells mediate immunity against fungal, viral, and bacterial infections (1, 13), participate in antitumor immunity in the mouse models (16), and are associated with poor prognosis in GI tract-associated cancers (33, 34). Tc17 cells can be pathological in autoimmune disorders like psoriasis, multiple sclerosis, and ulcerative colitis (12). We have previously shown that fungal vaccine-induced Tc17 cells mediate immunity and form stable, long-lasting memory without plasticity (13–15). The programming to become stable, long-lasting memory T cells happens during the expansion phase (35, 36), and metabolic adaptation is needed. In this study, we found that fungal vaccine-induced Tc17 cells portray resilience in mitochondrial dependence for metabolic needs despite they preferentially utilize glucose.

Tc17 cells were robust in cellular proliferation and showed higher active translation machinery, suggesting higher metabolism. Increased dependence on glucose for aerobic glycolysis is documented in several studies, and glycolysis is linked to activating CD8^+^ T cells to become efficient effector T cells. To meet immediate energy demand during activation and expansion, Tc17 cells were reliant on glucose at the outset, and inhibition of glucose metabolism by an analog, 2-DG, profoundly reduced the Tc17 cell responses. Additionally, in line with their metabolic phenotype (**Figure 1**), we found more pronounced defective responses than in Tc17 cells than Tc1 cells.

Since the T cells undergo significant changes during the expansion, including activation, differentiation, proliferation, and espousing effector functions, we evaluated the degree of glucose requirement in a phase-specific manner. Tc17 cells were quickly adapted to glucose sources for energy needs, and 2-DG treatment severely blunted their responses early in the expansion. However, the dependency on glucose by Tc17 cells was reduced during the later phases of expansion, despite their active metabolic needs for proliferation and protein synthesis. As expected, naïve T cells were largely unperturbed during the glucose deficiency. Experimental models show that high glucose favors Th17 cell responses and exacerbates autoimmune diseases (37, 38). Although we did not test the effect of superfluous glucose on type 17 CD8^+^ T cell differentiation (39) in our model system, our unpublished observations suggested a minor effect on RORγt expression in Tc17 cells.

Since glycolysis is linked to the activation of CD8^+^ T cells and performs effector functions through histone acetylation for epigenetic modifications (40) and our observations suggested predominant dependence on glucose for OXPHOS, we believe that lower level of glycolysis dependency during the expansion may be enough for their activation. Although OXPHOS drives a selective lactylation site and is enriched across naïve, activated, and memory T cell subsets (40), our observations suggest the differential metabolic pathways adopted by Tc17 and Tc1 cells. As overactivation of CD8^+^ T cells leads to functional exhaustion and clonal deletion (41), and inhibition of glycolysis promoted central memory CD8^+^ T cells (42, 43), the Tc17 cells’ adoption of both glycolysis and OXPHOS may help promote their stability, functionality, and survival while breaking the T-cell activation threshold. In support of this, the recent findings suggest the dependency of Th17 cells on OXPHOS to resist apoptosis (44) and drive pathogenic disease (45, 46). However, studies also suggest that promoting OXPHOS leads to biased differentiation of Tregs over Th17 (47, 48), suggesting a rheostatic effect of glycolysis-OXPHOS metabolism for T cell differentiation, functions, and longevity.

Our data showed that Tc17 cells are less reliant on glucose during the late phase of expansion despite their higher metabolic need at the peak of expansion, and 2-DG administration moderately but significantly affected the Tc17 responses. We hypothesized two possibilities. First, the glucose is used in a more efficient manner, i.e., utilizing the glucose-channeled OXPHOS pathway (49) and second, the cells adopt FAO/AAO pathway for energy generation through OXPHOS (50). Indeed, we found that Tc17 cells had higher mitochondrial mass, membrane potential, and showed higher mitochondrial dependency, suggesting an active role of mitochondria and an active OXPHOS metabolism. Further, we showed that the inhibition of mitochondria-driven ATP synthesis by oligomycin significantly reduced Tc17 but not the IFN-γ^+^ Tc1 cells. Additionally, when we compared the glucose/mitochondrial dependence, Tc17 cells showed a preponderant preference for mitochondria than Tc1 cells with concominant lower glycolytic capacity. Since glucose is still needed in the late phase of expansion, our study indicated that it is preferentially channeled to OXPHOS metabolism for efficient energy generation with some support from FAO/AAO.

Our study reveals the metabolic needs of CD8^+^ T cells for differentiation into Tc17 and Tc1 cells *in vivo*. Tc17 cells are highly reliant on OXPHOS despite the use of glucose and resilient to an aerobic glycolysis shift following activation. Adjuvants or modulators altering the metabolism of Tc17 cells may help bolster immunity or attenuate inflammatory diseases.

## Data Availability

The raw data supporting the conclusions of this article will be made available by the authors, without undue reservation.

## Supplementary material

Supplementary Figure 1 RORγt and IL-17F expression and proliferation of anti-fungal effector and early memory cells. Naïve 6- to 8- week-old WT or IL17a^Cre^R26R^eYFP^ mice were vaccinated with B.d. #55 as in **Figure 1**. At days 21 or 36 post-vaccination (PV), single-cell suspensions from dLNs and spleens were restimulated with αCD3/CD28 mAbs before staining for surface and intracellular cytokine staining followed by staining with anti-Ki-67, RORγt, or T-bet mAb. The gating strategy is shown in the upper panels. The percent IL-17^+^ subsets and RORγt/T-bet expression compared with IFN-γ^+^ subset as control **(A)**. Data indicate percent Ki-67^+^ naïve and cytokine-producing cells on D21PV **(B)** and D36PV **(C)**. Values are mean ± SD. n = 4–5 mice. Data are representative of at least two individual experiments. ***p≤ 0.001, and ****p≤ 0.0001.

Supplementary Figure 2 Glucose dependency of effector Tc17 cells for expansion. **(A)** *GLUT-1/2 expressions on effector CD8^+^ T cells*: Single-cell suspensions from dLNs of vaccinated IL17a^Cre^R26R^eYFP^ mice were stained direct ex vivo for surface markers and intracellular GLUT-1/2 and analyzed by flow cytometry. Data represents mean fluorescence intensities of GLUT-1/2 in CD44^lo^, CD44^hi^, and eYFP^+^CD8^+^ T cells. **(B)** Glucose utilization by effector CD8^+^ T cells: Vaccinated IL17a^Cre^R26R^eYFP^ mice were treated with either vehicle or glycolysis inhibitor 2-deoxy glucose (2-DG) intraperitoneally at days 5–15 PV. On D16PV, single-cell suspensions from dLNs were restimulated with αCD3/CD28 mAbs, stained for surface markers and intracellular cytokines and analyzed by flow cytometry. Data show frequencies eYFP^+^, GM-CSF^+^Tc17, TNF^+^, and GM-CSF^+^ cells gated on CD44^hi^ CD8^+^ T cells. Data are representative of two independent experiments. n= 4–5 mice/group. Values are mean ± SD. *p≤ 0.05, **p≤ 0.01,***p≤ 0.001, and ****p≤ 0.0001.

Supplementary Figure 3 Glucose utilization by activated CD8^+^ T cells during early and late phases of expansion: Vaccinated mice were administered with vehicle or 2-deoxy glucose (2-DG) intraperitoneally on indicated days. On days 7 or 21 PV, single-cell suspensions from dLNs were restimulated (as in **Figure 3**), stained, and analyzed by flow cytometry. Data indicate percent and total numbers of CD44^lo^ and CD44^hi^ cells gated on CD8^+^ T cells at days 7PV **(A)** and 21PV **(B)**. Data are representative of two independent experiments. n= 4–5 mice/group. Values are mean ± SD. *p≤ 0.05, **p≤ 0.01, and ***p≤ 0.001.

Supplementary Figure 4 OXPHOS utilization by activated CD8^+^ T cells: **(A)** Naïve CD8^+^ T cells were enriched and cultured with BMDCs pulsed with heat-killed yeast. On days 2–4 post-culture, cells were treated with vehicle or oligomycin. The cells were harvested on day 5, stained for cell surface markers, and analyzed by flow cytometry. Data show the percent and total numbers of CD44^hi^ CD8^+^ T cells. **(B)** The puromycin incorporation in CD8^+^CD44^hi^ cells in the experiments shown in **Figures 5B, C**. **(C)** The enriched CD8^+^ T cells from naïve mice were stimulated with plate-bound anti-CD3e MAb (10 μg/ml overnight) and soluble anti-CD28 MAb (2μg/ml) and polarized using cocktail of IL-6 (20ng/ml), TGFβ (2ng/ml), IL-1β (10ng/ml), anti-IFNγ (5μg/ml), and anti-IL-4 (5μg/ml) with a replacement at day 2 for 4 days with or without 2-DG (250μM)/oligomycin (2μM). On day 5, GolgiStop was added for 5 hrs and percent cytokine^+^ CD8^+^ T cells were analyzed by flow cytometry. Data are representative of two independent experiments except **(C)**. n= 4–5 mice/group. Values are mean ± SD. *p≤ 0.05, **p≤ 0.01, ***p≤ 0.001, and ****p≤ 0.0001.
