# Supplementary figures and images for: Effector Tc17 cells resist shift from OXPHOS to aerobic glycolysis
Source: Front Immunol. 2025 May 16;16:1571221. doi: 10.3389/fimmu.2025.1571221 (PMC12122523; doi:10.3389/fimmu.2025.1571221)

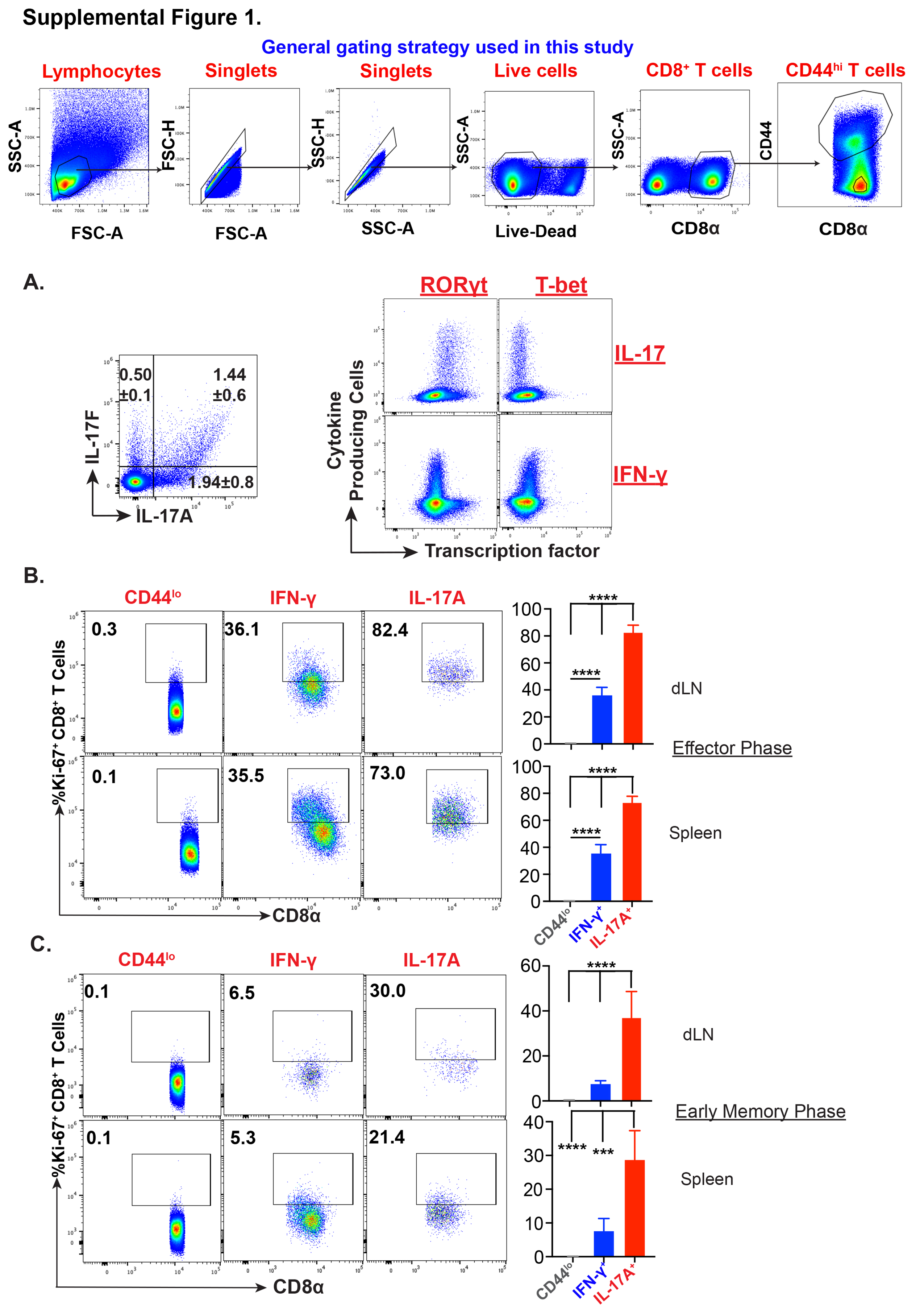

Supplement: Supplementary Figure 1 — RORγt and IL-17F expression and proliferation of anti-fungal effector and early memory cells. Naïve 6- to 8- week-old WT or IL17aCreR26ReYFP mice were vaccinated with B.d. #55 as in Figure 1 . At days 21 or 36 post-vaccination (PV), single-cell suspensions from dLNs and spleens were restimulated with αCD3/CD28 mAbs before staining for surface and intracellular cytokine staining followed by staining with anti-Ki-67, RORγt, or T-bet mAb. The gating strategy is shown in the upper panels. The percent IL-17+ subsets and RORγt/T-bet expression compared with IFN-γ+ subset as control (A). Data indicate percent Ki-67+ naïve and cytokine-producing cells on D21PV (B) and D36PV (C). Values are mean ± SD. n = 4–5 mice. Data are representative of at least two individual experiments. ***p≤ 0.001, and ****p≤ 0.0001. [file Image1.tif]

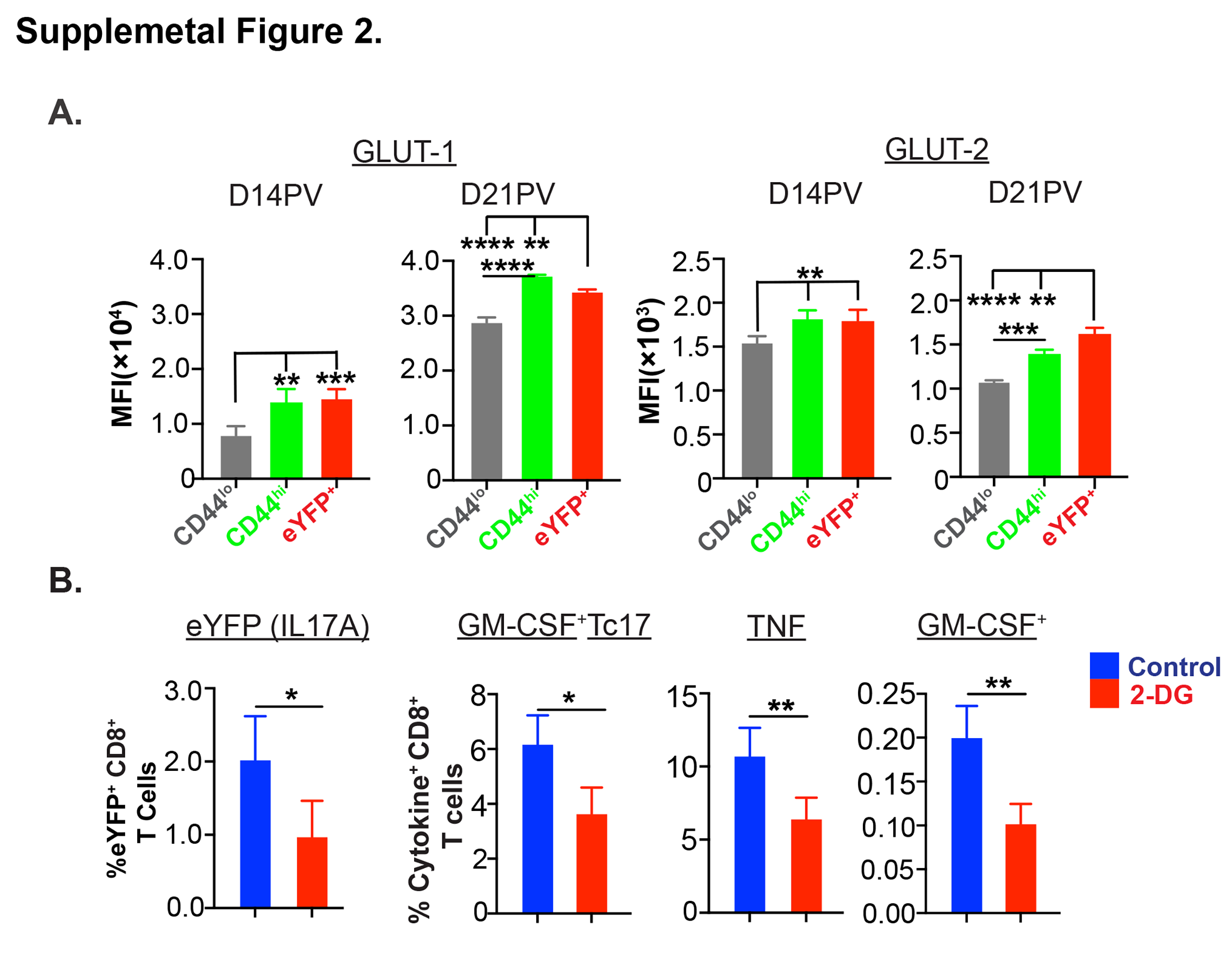

Supplement: Supplementary Figure 2 — Glucose dependency of effector Tc17 cells for expansion. (A) GLUT-1/2 expressions on effector CD8+ T cells: Single-cell suspensions from dLNs of vaccinated IL17aCreR26ReYFP mice were stained direct ex vivo for surface markers and intracellular GLUT-1/2 and analyzed by flow cytometry. Data represents mean fluorescence intensities of GLUT-1/2 in CD44lo, CD44hi, and eYFP+CD8+ T cells. (B) Glucose utilization by effector CD8+ T cells: Vaccinated IL17aCreR26ReYFP mice were treated with either vehicle or glycolysis inhibitor 2-deoxy glucose (2-DG) intraperitoneally at days 5–15 PV. On D16PV, single-cell suspensions from dLNs were restimulated with αCD3/CD28 mAbs, stained for surface markers and intracellular cytokines and analyzed by flow cytometry. Data show frequencies eYFP+, GM-CSF+Tc17, TNF+, and GM-CSF+ cells gated on CD44hi CD8+ T cells. Data are representative of two independent experiments. n= 4–5 mice/group. Values are mean ± SD. *p≤ 0.05, **p≤ 0.01,***p≤ 0.001, and ****p≤ 0.0001. [file Image2.tif]

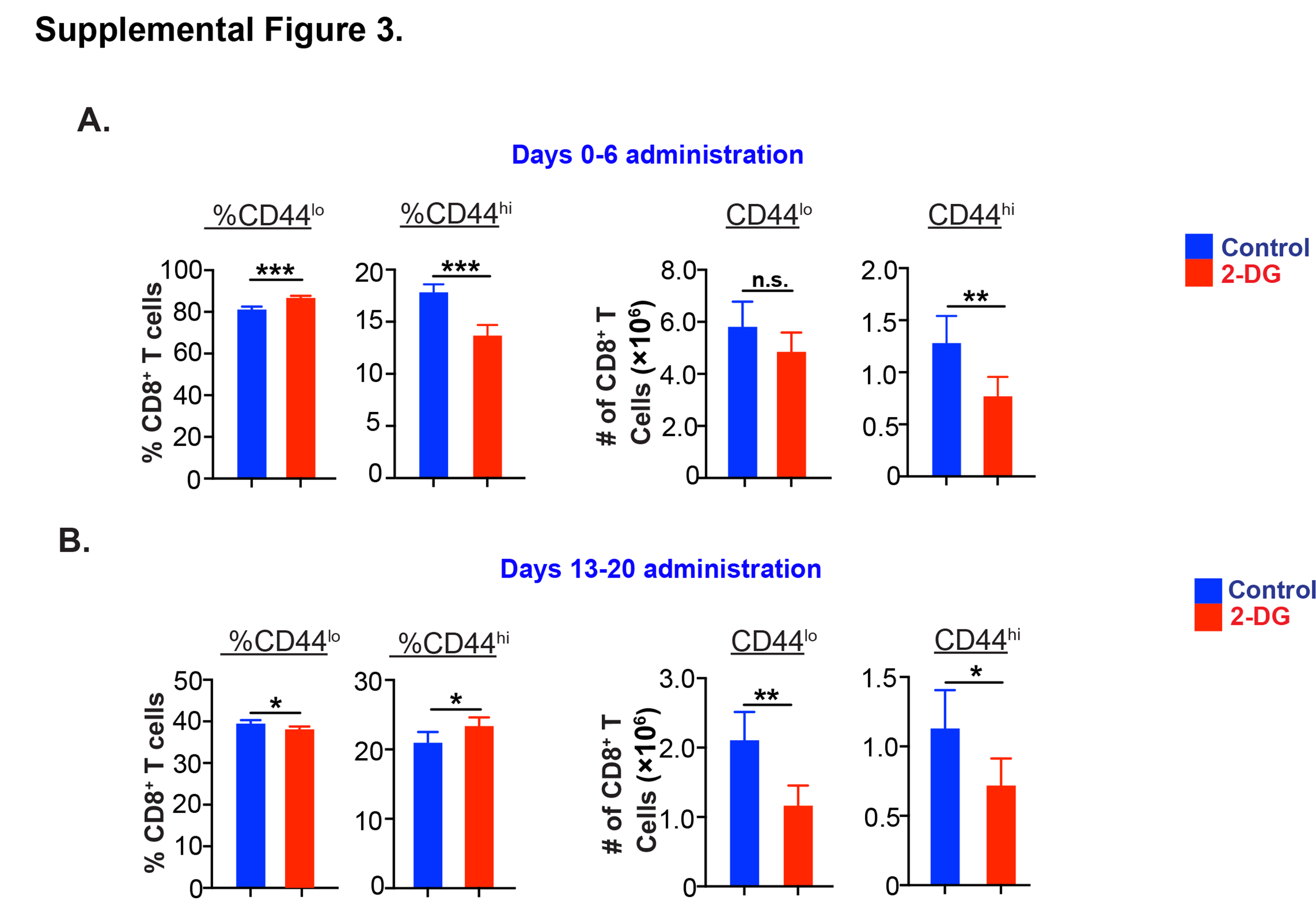

Supplement: Supplementary Figure 3 — Glucose utilization by activated CD8+ T cells during early and late phases of expansion: Vaccinated mice were administered with vehicle or 2-deoxy glucose (2-DG) intraperitoneally on indicated days. On days 7 or 21 PV, single-cell suspensions from dLNs were restimulated (as in Figure 3 ), stained, and analyzed by flow cytometry. Data indicate percent and total numbers of CD44lo and CD44hi cells gated on CD8+ T cells at days 7PV (A) and 21PV (B). Data are representative of two independent experiments. n= 4–5 mice/group. Values are mean ± SD. *p≤ 0.05, **p≤ 0.01, and ***p≤ 0.001. [file Image3.tif]

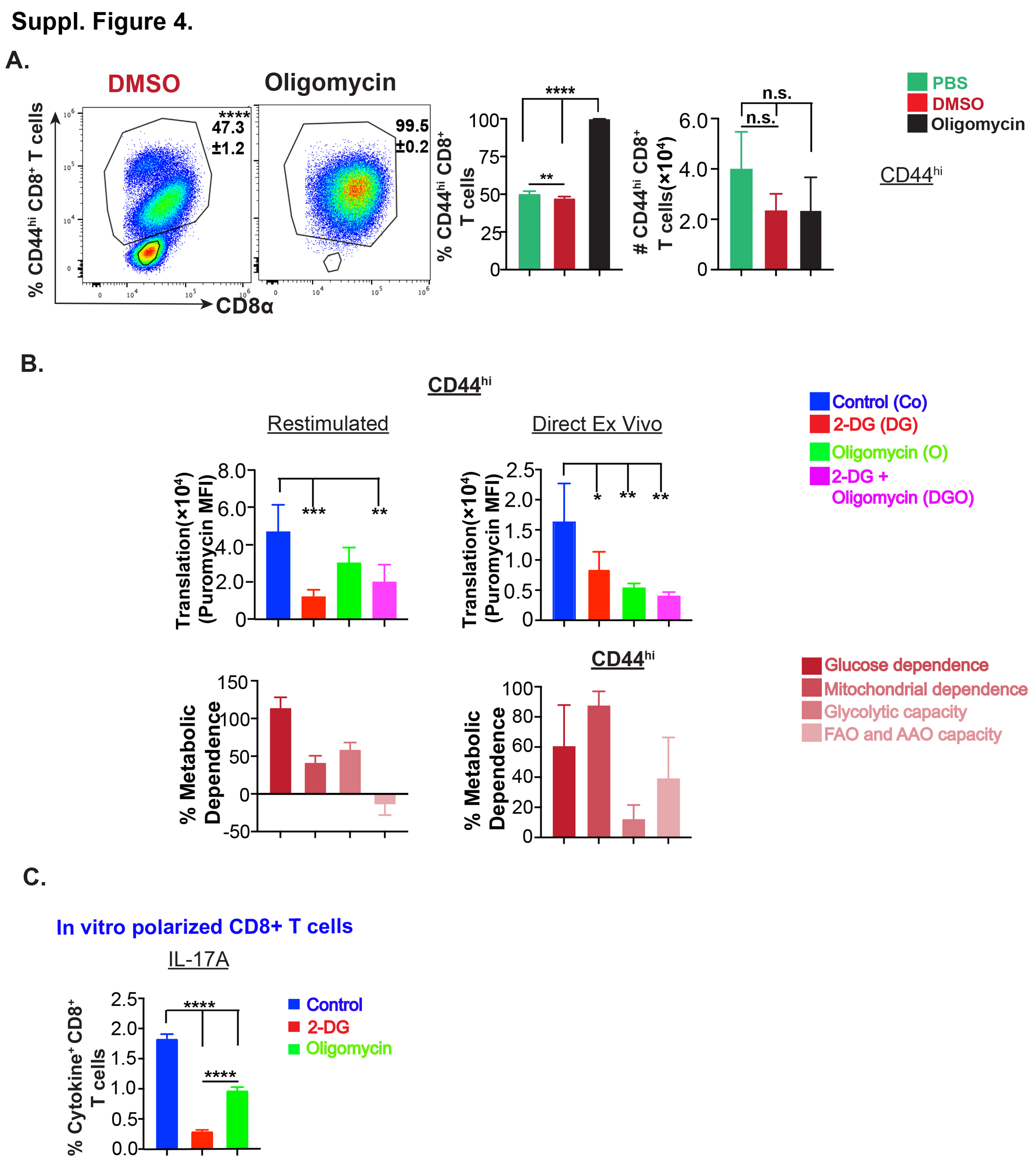

Supplement: Supplementary Figure 4 — OXPHOS utilization by activated CD8+ T cells: (A) Naïve CD8+ T cells were enriched and cultured with BMDCs pulsed with heat-killed yeast. On days 2–4 post-culture, cells were treated with vehicle or oligomycin. The cells were harvested on day 5, stained for cell surface markers, and analyzed by flow cytometry. Data show the percent and total numbers of CD44hi CD8+ T cells. (B) The puromycin incorporation in CD8+CD44hi cells in the experiments shown in Figures 5B, C . (C) The enriched CD8+ T cells from naïve mice were stimulated with plate-bound anti-CD3e MAb (10 μg/ml overnight) and soluble anti-CD28 MAb (2μg/ml) and polarized using cocktail of IL-6 (20ng/ml), TGFβ (2ng/ml), IL-1β (10ng/ml), anti-IFNγ (5μg/ml), and anti-IL-4 (5μg/ml) with a replacement at day 2 for 4 days with or without 2-DG (250μM)/oligomycin (2μM). On day 5, GolgiStop was added for 5 hrs and percent cytokine+ CD8+ T cells were analyzed by flow cytometry. Data are representative of two independent experiments except (C). n= 4–5 mice/group. Values are mean ± SD. *p≤ 0.05, **p≤ 0.01, ***p≤ 0.001, and ****p≤ 0.0001. [file Image4.tif]
